# A qualitative study exploring adolescents’ experiences with a school-based mental health program

**DOI:** 10.1186/s12889-015-2368-z

**Published:** 2015-10-21

**Authors:** Pernilla Garmy, Agneta Berg, Eva K. Clausson

**Affiliations:** Department of Health Science, Kristianstad University, Kristianstad, Sweden; Department of Clinical Sciences, Center for Primary Health Care Research, Malmö, Lund University, Lund, Sweden; Department of Nursing, Health and Culture, University West, Trollhättan, Sweden

**Keywords:** Adolescents, Depression, School-based program, Prevention, Cognitive behavior program

## Abstract

**Background:**

Supporting positive mental health development in adolescents is a major public health concern worldwide. Although several school-based programs aimed at preventing depression have been launched, it is crucial to evaluate these programs and to obtain feedback from participating adolescents. This study aimed to explore adolescents’ experiences with a -based cognitive-behavioral depression prevention program.

**Methods:**

Eighty-nine adolescents aged 13–15 years were divided into 12 focus groups. The focus group interviews were analyzed using qualitative content analysis.

**Results:**

Three categories and eight subcategories were found to be related to the experience of the school-based program. The first category, intrapersonal strategies, consisted of the subcategories of directed thinking, improved self-confidence, stress management, and positive activities. The second category, interpersonal awareness, consisted of the subcategories of trusting the group and considering others. The third category, structural constraints, consisted of the subcategories of negative framing and emphasis on performance.

**Conclusions:**

The school-based mental health program was perceived as beneficial and meaningful on both individual and group levels, but students expressed a desire for a more health-promoting approach.

**Electronic supplementary material:**

The online version of this article (doi:10.1186/s12889-015-2368-z) contains supplementary material, which is available to authorized users.

## Background

Supporting positive mental health development in adolescents is a major public health issue worldwide. Adolescent depression is associated with impaired academic performance, social difficulties, abuse and neglect, and substance use [1]. Although adolescence is the healthiest period in life [2], the many positive and negative aspects of normal teenage development have been described as an emotional roller coaster [3]. A large group of youth is at risk for depression, which differs from normal teenage mood swings. The prevalence of adolescent depression is approximately 4–8 % worldwide [1, 4], and adolescent depression is more common among females than among males [5]. However, many depressed adolescents are reluctant to seek professional help for mental illness and are therefore not diagnosed [6], or they do not reach the diagnostic threshold for major depressive disorder but still have symptoms that may have long-term clinical and social implications, such as school failure, loss of confidence, and isolation [1]. Approximately 60 % of adolescents with depression have recurrent episodes in adulthood [7]; therefore, the need for early prevention of depression in adolescents has been emphasized. Depression represents a major cause of morbidity and disability worldwide, and according to the World Health Organization, it is the fourth-leading cause of disease burden [4].

Health promotion, according to the Ottawa Charter [8], includes providing a supportive environment and opportunities for making healthy choices. Universal prevention programs have been advocated because they have a greater reach than selected or indicated programs do [9]. The majority of universal prevention programs targeting depressive symptoms are based on cognitive-behavioral therapy (CBT) [10]. Several school-based programs aimed at preventing depressive symptoms in adolescents have been launched [11]. However, it is of crucial importance to evaluate these programs further and to obtain feedback from the voices of participating adolescents [12]. The Ottawa Charter [7] states that health promotion strategies and programs should be adapted to the local needs and resources of individual countries to account for differing social, cultural, and economic systems.

In Sweden, students have 9 years of compulsory schooling. Schools must follow national learning curricula, and school administrations can include extracurricular subjects in the class schedule, such as a school-based depression prevention program. Schools are required by law to employ school health staff, such as a school nurse and a school social worker [13]. A growing awareness of the increase in mental health problems among adolescent girls in the late 1990s led the council in Stockholm to charge the Center for Public Health with developing a school-based intervention to prevent stress and depressive symptoms. The thoroughly evaluated cognitive-behavioral program Coping with Stress (CWS) [14–17] was modified and adapted for the Swedish setting and was called DISA (Depression in Swedish Adolescents). Whereas CWS is an indicated program targeting individuals with risk factors for developing depression, DISA is a school-based, universal program targeting adolescents approximately 13–15 years old. This age group was chosen because adolescents at this age are considered sufficiently mature to grasp the theory of the program and because depression rates in this age group have been increasing. However, because DISA is a prevention program, the intervention is intended to be implemented before depression arises [18]. Originally targeting girls only, DISA is still primarily administered to girls. Today, the program is also offered to boys in some Swedish schools.

If a school chooses to use the DISA program, school health staff and/or teachers are trained to be tutors in a three-day course. Typically, two tutors conduct one course with approximately 8–15 participants. The course may be voluntary or compulsory.

Although research on the DISA program has shown positive effects in terms of a decrease in depressive symptoms [18, 19], the program has also been criticized for its pathogenic focus and the risk of stigmatization in being offered to girls only [20–22]. Nevertheless, Wickström [20] found that adolescent girls perceived attending the DISA program as meaningful, although the students and tutors did not strictly follow the manual. This finding was confirmed in a focus group study in which DISA tutors claimed to strike a balance between strictly following the manual and meeting students’ needs [23]. In another study [19], written comments on the program showed that the majority of students appreciated the DISA course because they learned a new way of thinking and became better acquainted with others in the group. These contradicting findings regarding the strengths and weaknesses of the DISA program suggested a need for a deeper understanding of students’ opinions about the course. To the best of our knowledge, this is the first focus group study with both males and females attending the DISA program. This study aimed to explore adolescents’ experiences participating in a school-based mental health program.

## Methods

A qualitative focus group design was chosen to capture the experiences of adolescents who had participated in a school-based mental health program [24]. The study was conducted and reported in accordance with the RATS (Relevance, Appropriateness, Transparency, Soundness) guidelines for qualitative research [25]. Ethical approval for the study was obtained from the Regional Ethical Review Board in Lund (2012/462) prior to recruitment. After receiving written information about the study and its voluntary nature, the participating adolescents and their parents provided written informed consent.

The program consisted of 10 weekly manual-based sessions, each of which was 90 min long with 7–18 students. The program is based on cognitive-behavioral techniques for changing negative thoughts, communication training, problem-solving strategies, exercises to strengthen social skills and social networks, and increased participation in health promotion activities. DISA is a manual-based program, and every session is designed around a certain topic; see Table 1. The tutors were school social workers, school nurses, and teachers who had completed a three-day DISA tutor training course.

**Table 1:** DISA is a manual-based program, and every session is designed around a certain topic

Session	Theme
1	Getting to know each other, program rules
2	Coping with stress
3	Identification of negative thoughts
4	Positive thinking
5	Changing negative thoughts to positive thoughts
6–8	Identifying negative thoughts
9	Communication practice
10	Maintaining well-being

Treutiger and Lindberg 2012, p. 64 [18]

### Sample

The study was conducted in four municipalities in rural and urban areas of southern Sweden. The sample consisted of adolescents aged 13–15 (grade 8). The school-based mental health program DISA was conducted in 8 of the 11 schools in the included area. These eight schools were contacted, but two of them declined participation because of a lack of time (it was not possible to allow students to participate in a focus group interview during the school day). At the remaining six schools, 12 focus group discussions were conducted with a total of 89 students (25 % males) whose median age was 14 (range 13–15). Each focus group represented one DISA course, met once, and consisted of 3–11 participants; see Table 2. In seven focus groups, all of the DISA course participants engaged in the discussion, but in five focus groups, a few of the original DISA course participants declined participation (*n* = 7) or were absent the day of the focus group discussion (*n* = 9). These 12 focus groups were considered to be sufficient because no new content was revealed in the last set of interviews.

**Table 2:** Focus group, sex, school, and number of participants

Focus group (number)	Sex	School (name)	Participants (n)	Age (years)
1	Female	A	6	14
2	Female	B	7	14–15
3	Female	C	5	14–15
4	Female	D	6	14–15
5	Female	E	8	13–15
6	Female	E	11	14–15
7	Female	E	11	14–15
8	Male	F	9	14
9	Female	F	10	14
10	Male	F	5	14
11	Male	F	8	14
12	Female	F	3	14

### Data collection

Focus group interviews were conducted to capture the various experiences of the adolescents [24]. The key motivation for conducting a focus group discussion is that it promotes reflection on different opinions and the further articulation of thoughts. All focus group interviews were conducted from March 2014 to February 2015 at the adolescents’ schools during the school day, and the duration of the interviews was 30–70 min. The first author moderated the interviews, and the last author served as an observer (for practical reasons, the last author observed only the first three sets of focus group interviews). The role of the moderator was to maintain a focused discussion and to ensure that everyone participated. The observer focused on the group interactions and took field notes. The groups were guided using a semi-structured, open-ended topic list with questions such as “What are your thoughts on the DISA course?” (see Additional file 1). To ensure that the discussion focused on the specific question, the moderator used visual aids in the form of A4 pages with topics that the students were asked to discuss. For example, one A4 page read “The DISA course,” and another read “Adolescent health.” During the interviews, further probing questions were asked about each discussion topic to elicit deeper responses. The focus group interviews were audio-recorded and transcribed verbatim by the first author.

### Analysis

The transcribed text was analyzed using qualitative content analysis [26]. Content analysis enables the analysis of large quantities of data and focuses on variations in views [27]. Qualitative content analysis comprises descriptions of the concrete content and interpretations of the abstracted content while maintaining focus on subjects’ experiences [28]. The transcripts were read repeatedly to achieve immersion and to obtain a sense of the whole picture. Sections of the text related to adolescents’ experiences participating in a school-based mental health program were combined into one text to form a content area. This text was divided into meaning units (*n* = 478), which were then condensed, abstracted, and labeled with codes. The context as a whole was considered during the condensing and coding process. The codes were compared on the basis of differences and similarities and sorted into three categories and eight subcategories. The first author conducted the first coding. The second and third authors independently coded two interviews each, and the three authors subsequently met and discussed the coding until they reached consensus. All authors reflected on and discussed the codes, categories, and subcategories throughout the analysis process to increase the level of trustworthiness [26].

## Results

The students’ experiences with the school-based mental health program DISA are presented below and illustrated by quotations from different focus group interviews. The results are presented in three categories: (A) intrapersonal strategies, (B) interpersonal awareness, and (C) structural constraints (see Table 3). Each category includes two to four subcategories. Most adolescents perceived the need for a course such as DISA, and they believed that the age of 14 or 15 was a good age for such a course because adolescents feel substantial pressure at this age and are old enough to understand the message of the course. The results revealed the adolescents faced many challenging demands and that a course such as DISA made it easier for them to meet these demands, such as challenges related to their appearance, dress and performance. The most important part of the program seemed to be the conversations. Good conversations were characterized by trust and concerned deep matters, but laughter and humor were still present.

**Table 3:** Categories and subcategories

Categories	Subcategories
A. Intrapersonal strategies	A.1 Directed thinking
	A.2 Improved self-confidence
	A.3 Stress management
	A.4 Positive activities
B. Interpersonal awareness	B.1 Trusting the group
	B.2 Considering others
C. Structural constraints	C.1 Negative framing
	C.2 Emphasis on performance

### Intrapersonal strategies

This category describes the intrapersonal strategies that students learned in the course. The subcategories identified were directed thinking, improved self-confidence, stress management, and positive activities. Most adolescents described a need for a course such as DISA at school, and the majority claimed that the course had provided them with beneficial strategies on an individual level.

#### Directed thinking

The subcategory of directed thinking describes how the course helped the students to think more positively. According to the students, they learned to identify negative thoughts and turn them into positive thoughts and to try to think positively after having negative thoughts. This new ability made them feel happier and more alert: *“You think more positively and so…changing negative thoughts into positive thoughts. One feels happier, more positive…more alert”* (male, focus group 8). The students gave the example that if they felt too bored to attend school, they could think of the pleasant experience of meeting their friends and then feel better. Viewing things in a new light was highlighted as significant. However, the students also stated that identifying negative thoughts made them feel negative during the class. The link among thoughts, feelings, and behavior was mentioned. The students mentioned the possibility of influencing one’s thoughts and emotions with one’s choice of actions. The students indicated that they practice this on a daily basis now because of their training through talking and course exercises:

- *It’s probably the conversations [that have a preventive effect]*
- *But there are some good exercises too, which are good, about the triangle [the link between thoughts, feelings and behavior], for example, which was good…*
- *It is preventive for the future, and you think of it anyway* (females, focus group 4)

#### Improved self-confidence

Improved self-confidence was another frequently mentioned aspect: *“I actually learned a lot from it, self-confidence, empathy, and such things”* (male, focus group 10). The effect of being satisfied with oneself and not attempting to be someone else was highlighted. One participant expressed her feelings as follows: *“I feel much more confident in myself. You are good as you are. You don’t need to be someone else”* (female, focus group 1). In an experience emphasized as a positive experience in several groups, the group members had to write positive comments about one another. Knowing what the other students in the group liked about themselves was perceived as a significant component:

- *We had to write what we thought about each other in the group*
- *It was fun, and then you should say it yourself, kind of “I am…”*
- *It felt a little egocentric*
- *But it was good*
- *You seldom compliment yourself*
- *Often girls receive more negative comments than kind ones* (females, group 4)

The role of the tutor was also regarded as an important and positive factor. One participant summarized this view as follows: *“[The tutor] has picked up, sort of, things that we are good at, sort of tried to strengthen our self-confidence”* (female, focus group 12).

#### Stress management

The stress management subcategory describes how the students were able to manage stress and consider difficult situations in new and different ways after the course. The students also expressed that they could use what they had learned in future stressful events. According to the students, the insight that thoughts, emotions, and behavior are linked together enabled them to change their behavior to create less stressful conditions: *“Thoughts, actions and feelings are linked together.… If you change your behavior, then your emotion might change automatically and then your thoughts”* (female, focus group 4). Students involved in sports mentioned that techniques for coping with situations that were not going as expected (e.g., avoiding the event, changing the event, or changing the way they reacted to the event) were good to use in sports. The students also stated that they used the DISA techniques before stressful exams. By telling themselves that they would perform well on the exam, they were able to manage such situations better.

The students appreciated the course for helping them to think before talking or acting and to think of the consequences of their words and actions. One participant summarized this view as follows: *“Thinking before doing something and not just acting before you have thought about it”* (male, focus group 11). The students also stated that the course helped them to manage their anger and reduce violence, as illustrated by the following discussion:

- *It was useful for me. I can get angry sometimes.*
- *Me too.*
- *Before, I didn’t think before I acted…for example, I fought a lot before* (males, focus group 10)

#### Positive activities

The adolescents described how the DISA course helped them to focus more on things that they truly enjoyed and to choose positive activities that were good for them. They learned what was good for them and what made them happy and found that they wanted to do more of these activities: to feel good, they had to engage in activities that that they enjoyed. Examples of activities that they had begun to participate in or to spend more time doing differed among individuals, but listening to music, relaxing, writing, and breathing calmly were frequently mentioned: *“I love to write, and I have started to write more since the program”* (female, focus group 2). Other students claimed that they learned of the benefits of being physically active; even if they did not want to be active, they could still try to be active because they knew that such activity is good for them: *“I do sort of the same thing as before, but I am outdoors more often, going out with the dog”* (female, focus group 1). Being more socially engaged with their families and neighbors was also mentioned. One exercise that they appreciated was listing what they liked to do and examples of things that would make them feel better, such as taking a bath, walking, or reading a book. Making such a list was experienced as a nice and comforting activity, counterbalancing the parts of the course that focused more on negative matters.

### Interpersonal awareness

The second category describes interpersonal awareness, implying a group-level impact during and after the course. The subcategories identified were trusting the group and considering others. Most of the adolescents referred to positive experiences on an interpersonal level.

#### Trusting the group

The subcategory of trusting the group describes experiences of increased group cohesion during and after the course. By openly speaking about their problems, the students learned that they were not alone in having negative thoughts and doubts about themselves, and this discussion made it easier for them to manage their negative thoughts and doubts. The group became closer through their discussions with one another: *“And you have become closer to your classmates”* (male, focus group 11). In one focus group, students mentioned that before the course, they had different friendship groups, whereas after the course, they could spend time with anyone in the group:

- *Before, we were split into three groups of friends, but now there is better group cohesion, just like the boys. Everyone can be with everyone.*
- *We have come a little bit closer to each other than we were before the course* (females, focus group 1)

Another good experience from the course that improved trust in the group was the exercise in which the participants had to write down something positive about the others in the group. The students appreciated becoming acquainted with new friends in the course: *“It has been good; you learn to know more people, and I’ve found it cozy”* (female, focus group 7). The students liked when the tutors arranged the class to ensure that they would sit beside someone with whom they did not often speak outside of class. The students thus became acquainted in different ways and could see one another from new perspectives. For example, a student might have thought that someone was tough and cool but subsequently realized that he or she was actually a kind person. The communication exercises in the program contributed to this realization. In one course activity, the students each brought something that symbolized something important to them and shared it with the others in the group. They appreciated the ability to share something important with their classmates and to learn new things about one another.

Most students reported that it was good for the groups to be separated by sex. Both girls and boys claimed that they were able to speak more freely because of this separation. However, they did not think the gender of the tutor was relevant; rather, the tutors’ personality was the most important factor. Both boys and girls stated that the group was calmer when the sexes were separated. Although they felt that it was beneficial to mix boys and girls in other subjects, they thought that the separation was good in a course such as DISA that involves discussing emotions.

#### Considering others

The subcategory of considering others describes the knowledge gained from the course related to awareness of the emotions and behavior of other people. The students thought that their level of empathy improved because they learned how other people live. The students claimed that they learned to be more tolerant and considerate as a result of this insight: *“And you learn how to tolerate”* (male, focus group 11). The students also received tools to help other people, as illustrated in the following discussion:

- *I would have said to [my little sister] anyway to take the course because I feel that I understand other people much better now. Because you get to learn about how others have it.*
- *You have more consideration; you sort of get insight.*
- *Yes, you also learn how to help that other person* (females, focus group 3)

Students who were active in sports claimed that they learned to think more about how to behave with one another and that all players on a team are equally important. In one course exercise involving active listening, one student speaks while the other students alternate between being disinterested and being alert. The students stated that they learned a great deal about how much the listener influences the speaker. They learned how frustrating it is to be ignored. One lesson from this exercise involved looking people in the eyes and not speaking only about oneself. Another example of changed behavior was beginning to speak more to the grocery store cashier rather than using their mobile phones, as they realized that this person needed to be respected: *“I have started to talk more with the cashiers at the grocery store and not using my mobile while shopping because you really meet another human being there, a cashier”* (female, focus group 3).

### Constraining structures

The third category describes the constraining structures. The subcategories identified were negative framing and emphasis on performance. The students articulated the desire for a more positive and health-promoting focus as well as more time for discussions rather than written course assignments.

#### Negative framing

The subcategory of negative framing describes a frequently mentioned complaint regarding the major focus on negative thoughts in the course. The students described the manual as focusing on problems rather than opportunities. The students found it difficult to identify their negative thoughts and then to hold onto them and count them. They said that their thoughts were originally positive, but they felt as if they were forced to bring forward negative thoughts and feelings of sorrow. Small problems became larger than they should have been. The students stated that they felt more stress when they had to think of such matters. At schools where the course was offered to girls only, some girls interpreted this framing as the school expecting girls to have problems: *“Yes, there is [a need for a course like DISA], but it is strange that it takes for granted that girls need it but not the boys.…It is taken for granted that girls will feel bad”* (female, focus group 3). The students articulated the desire for a more positive focus. They stated that a course focused more on joy would enable them to better grasp the serious content. Rather than focusing on how to eliminate negative thoughts, they believed that it would be better for the course to focus on how to find and retain positive thoughts: *“Partly it helped. But I think that it would have worked better if instead of focusing on how to get rid of the negative thoughts, you had focused more on how to get positive thoughts. In that case, you might have been a little bit happier, and then it would have worked better”* (female, focus group 6). Some students even reported that they felt worse while attending the course, but only until the session ended. When they left the classroom and met their friends, the students felt normal again, but during the course, they found themselves feeling unhappy or depressed for a while. They reported that they came to the course feeling positive and happy, but they began to feel unhappy when being asked to search for their negative thoughts and feelings during the course.

#### Emphasis on performance

The subcategory of emphasis on performance indicates a desire for more discussions rather than the large amount of writing that needed to be completed during every session: *“[I wished] it was deeper, not just sitting and filling out paperwork”* (female, focus group 9). The students considered the discussions (not the writing assignments) to be the crucial part of the course. According to the students, learning to know themselves was best accomplished by talking, not by writing. They indicated that the writing assignments were overly superficial; it was during the conversations that depth emerged, as illustrated in the conversation below:

- *The bad thing was that you sort of wrote the same thing every week.*
- *You actually don’t need to write. I think that we are here to learn to know oneself, yes, talking, not writing. You get bored and you can’t concentrate* (males, focus group 11)

The students suggested that another method of addressing serious matters could involve more practical exercises and games where they could move around and perhaps engage in role-playing. Some students wanted to work more on what the group needed rather than working on what the manual focused on for a certain session. The students wanted to work with issues that were important to them rather than the issues specified in the manual. However, some students found the manual to be a good support for the tutors. One student noted that class discussions outside of DISA could be less focused and, therefore, less meaningful: *“It would not have been good [without the manual] because first we would talk a lot, and then we would not have anything to say, and then we would have started to talk about something else”* (female, focus group 4). However, some students stated that the manual did not target their age group. They found the exercises to be irrelevant and artificial and not related to youths their own age: *“I think some of the issues in the course were not related to us.…It would have been better if it had been related to girls our age”* (female, focus group 5).

## Discussion

The main finding was that the school-based mental health program DISA was perceived as beneficial for both intrapersonal strategies and interpersonal awareness; however, structural constraints regarding the negative framing of the course and the emphasis on performance were also identified.

The intrapersonal strategies include directed thinking, improved self-confidence, stress management, and positive activities. Interpersonal awareness concerns trusting the group and showing consideration for others. According to many students, they achieved a better understanding of the links between thoughts, feelings, and actions, and in some cases, this understanding led to changes in their ways of thinking, improved self-confidence, new behaviors (for example, engaging in more activities that they enjoyed), and a better understanding of others. Improved group cohesion was another course outcome that was appreciated in several groups. The talking among students during the course also facilitated interaction outside the course. This result is consistent with the findings from an earlier study [19] that summarized the written comments of students in two categories: achieving a new way of thinking and becoming better acquainted with one another. This finding is also consistent with the work of Shochet et al. [29], who investigated adolescents’ experiences with another cognitive-behavioral-based depression prevention program. The authors found program benefits such as improved interpersonal relationships, improved self-regulation, and more helpful cognitions (ibid.).

The program benefits in our study were influenced by the constraining structures of the DISA program, particularly the negative framing of the program and the emphasis on performance. The DISA program is delivered in the classroom to a primarily healthy population of students; in contrast, the original course can be traced back to the indicated program CWS [14], which is designed for individuals with some depressive symptoms. Changing the target group for a prevention program could cause problems. In a previous study, Gunnarsson [22] questioned the increasing trend of health-promoting activities at schools, arguing that they are counterproductive because they imply that students are not good enough as they are and that their competence needs to improve to be able to handle the changing and difficult world. However, the majority of the students in the present study appreciated the DISA program and claimed that the course had helped them to improve their self-confidence. School has been regarded as an appropriate setting for depression prevention programs because this setting allows interventionists to target individuals at a young age—early prevention has benefits—and reach all individuals in a population [30]. This view is also consistent with the Ottawa Charter, in which school is regarded as a supporting environment [8]. Taylor et al. [31], however, emphasized how challenging it can be to deliver school-based mental health programs under everyday conditions because schools are complex and busy organizations with many competing demands in terms of time and resources.

The mental health intervention implemented in this study was delivered to males and females separately. Nearly all adolescents identified this separation as an advantage, but in the schools that offered the intervention to girls only, some girls criticized this policy; they perceived the schools as assuming that females feel bad while males do not. This criticism was also found in a study by Lindholm and Nelson [21]. Whereas the American intervention CWS [14] targets both sexes, the Swedish modified version was originally developed to target adolescent girls because of the increase in self-reported mental health complaints in this population in the 1990s [18]. Although being female is a risk factor for depression [5], the question remains as to whether such a targeted program could stigmatize rather than help girls. In the work of Rapee et al. [32] investigating adolescents’ reactions to universal and indicated prevention programs for depression, the authors found that despite the programs’ association with greater perceived stigma, they were also evaluated more positively by the participants.

### Limitations

The strength of focus group discussions is that different opinions and views can be reflected and discussion can result in further articulation of thoughts and ideas [24]. Such benefits were obvious in some groups in this study, especially in groups where the students became well acquainted well during the DISA course and groups where the atmosphere was relaxed and safe. However, in a few groups, the students seemed to be highly aware of one another to the extent that they were afraid of doing or saying something wrong or inappropriate in the group. In focus group discussions, consensus is not the goal; rather, the aim is to gather a variety of experiences. However, in the early adolescent phase at 13–15 years old, peers are extremely important, and being accepted and part of the group is crucial. Therefore, it might be difficult to capture different views from adolescents who strive to be similar to others in the group. For this reason, conducting individual interviews might have been beneficial. However, both the interviewer and the moderator were school nurses who have more than 10 years of experience each in talking with adolescents, and we sought to create a comfortable and relaxed atmosphere. To allow students to speak without thinking that they were divulging private information, the questions were often reworded into sentences such as “How would it have been, not for you but for a friend of yours or for someone else at this age?” When the students began to talk about someone else, the discussion could slowly enter a more personal sphere to avoid embarrassing the participants. Moreover, the ideal size of a focus group is five to eight participants [24], but one of our groups had only three participants. The discussion proceeded smoothly in this group, but two of our groups had 11 participants, and we observed that it was difficult for all participants to share their experiences in such large groups. Furthermore, the moderator had to ask them several times not to lean over and whisper to the next person but, instead, to talk to the group as a whole. The moderator had the responsibility of providing a safe atmosphere, and although the groups differed in how openly they spoke, we found that the conversation was friendly and that the adolescents were respectful of one another.

The question of transferability must be considered. The authors reflected on and discussed every step of the analysis process until they reached consensus in their interpretation. In this study, the voices of both males and females were heard; however, most participants were girls, as the DISA course is typically offered to females only. The participants were recruited from six schools in four municipalities; however, all the males in the study came from only one school. A few students declined participation in the focus group discussion; thus, we do not know the opinions of these students. However, the participating students expressed a variety of views about the DISA program, both positive and negative. Given that schools of different sizes in rural and urban municipalities were represented and that a variety of views were articulated, we argue that these findings could be valid in other settings with adolescents in this age group who have attended a cognitive-behavioral mental health intervention such as the DISA course.

## Conclusions

The school-based mental health program was perceived as beneficial and meaningful, although constraining structures were found. Some students considered the program manual to be incomprehensible and useless, whereas others vividly described how they had gained valuable knowledge from the course theory. One might ask whether it is possible to deliver a program that would satisfy all participants. However, in nearly all focus groups, the participants were vocal about their perception that the program focused too much on negative matters. This reaction might have arisen because the program is a depression prevention program rather than a health promotion program. Because of the program’s universal character, it might be more appropriate if it had a more positive, health-promoting focus that might be more widely accepted by this age group. Such a focus might also be beneficial for other school-based CBT depression prevention programs and for future program development.

## Additional file

Additional file 1:
**An overview of the semi-structured questions used during the focus group.** (DOCX 13 kb)

## Abbreviations

CBT: Cognitive behavioral therapy
CWS: Coping with stress course
DISA: Depression in Swedish adolescents
