# Supplementary material for: A qualitative study exploring adolescents’ experiences with a school-based mental health program
Source: BMC Public Health. 2015 Oct 21;15:1074. doi: 10.1186/s12889-015-2368-z (PMC4618132; doi:10.1186/s12889-015-2368-z)
Supplement: Additional file 1: — An overview of the semi-structured questions used during the focus group. (DOCX 13 kb) [file 12889_2015_2368_MOESM1_ESM.docx]

**Additional file 1:** An overview of the semistructured questions used during the focus group. The text in brackets [] refers to the visual aids in the form of A4 pages with topics that the students were asked to discuss.

1. What are your thoughts on the DISA course? [DISA course]
2. Tell me about the DISA tutor. [DISA tutor]
3. How are adolescents today? [Adolescent health]
4. Tell me something about the practical issues. [Practical issues (locality, schedule…)]
5. What about having the DISA course for girls or boys separated, or mixed? [Girls – boys – mixed]
6. What about having the course as a voluntary one or mandatory? [Voluntary or mandatory?]
7. Can you give examples of something especially significant? [Examples of something significant]
8. Can you give examples of something difficult or less good? [Examples of something difficult / less good]
9. Has the DISA course had an impact on your way of thinking? [Has the DISA course had an impact on your way of thinking?]
10. Has the DISA course had an impact on your way of acting? [Has the DISA course had an impact on your way of acting?]
11. Would you recommend any changes in the DISA course? [Change DISA?]
12. Are there any aspects we did not discuss that you would like to mention? [?]
